# Insecticidal Activity of Essential Oils from *Syzygium aromaticum* and *Foeniculum vulgare* Var. *dulce* and Their Major Compounds Against *Spodoptera frugiperda* and Physiological Selectivity for *Trichogramma pretiosum*

**DOI:** 10.3390/plants15193058

**Published:** 2026-10-07

**Authors:** Brenda C. F. Braga, Maria G. Venâncio, Júlia A. C. Oliveira, Karolina G. Figueiredo, Dejane S. Alves, Geraldo H. Silva, Denilson F. Oliveira, Geraldo A. Carvalho

**Affiliations:** 1 Department of Entomology, Federal University of Lavras—UFLA, Lavras 37203-202, MG, Brazil; brendafreirebraga@gmail.com (B.C.F.B.); maria.venancio2@estudante.ufla.br (M.G.V.); julia.assuncaooliveira@hotmail.com (J.A.C.O.); kgfigueiredo17@gmail.com (K.G.F.); 2 Federal Technological University of Paraná—UTFPR, Santa Helena 85892-000, PR, Brazil; dejanealves@utfpr.edu.br; 3 Institute of Exact and Technological Sciences, Federal University of Viçosa—UFV, Rio Paranaíba Campus, Rio Paranaíba 38810-000, MG, Brazil; geraldohumbertosilva@gmail.com; 4 Department of Chemistry, Federal University of Lavras—UFLA, Lavras 37203-202, MG, Brazil; denilson@ufla.br

**Keywords:** botanicals insecticides, integrated pest management, physiological selectivity

## Abstract

*Spodoptera frugiperda* (Lepidoptera: Noctuidae) causes significant losses in major crops and is mainly controlled with chemical insecticides and genetically modified plants; however, improper use of these strategies has led to the development of resistant populations. We evaluated the insecticidal activity of essential oils (EOs) and major compounds of *Syzygium aromaticum* (Myrtaceae) and *Foeniculum vulgare* var. *dulce* (Apiaceae) against *S. frugiperda*, and their selectivity toward *Trichogramma pretiosum* (Hymenoptera: Trichogrammatidae). EOs and major compounds were topically applied to caterpillars using a microsyringe. Chromatographic analysis revealed that the major compounds of *S. aromaticum* were eugenol (76.2%) and (*E*)-caryophyllene (19.9%), while (*E*)-anethole (83.35%) was the major compound of *F. vulgare* var. *dulce*. The EOs of *S. aromaticum and F. vulgare* var. *dulce* were toxic to *S. frugiperda* (LD_50_ = 54.2 and 41 µg/caterpillar, respectively), compared with 0.03 µg a.i./caterpillar for chlorpyrifos, with (E)-anethole accounting for most of its insecticidal activity. The EOs also adversely affected larval development, fecundity, and life table parameters. Additionally, the EO of *S. aromaticum* was harmful to adult *T. pretiosum* females, whereas *F. vulgare* var. *dulce* EO and (E)-anethole were harmless to slightly harmful to the parasitoid’s immature stages, highlighting their potential for selective control of *S. frugiperda*.

## 1. Introduction

The fall armyworm, *Spodoptera frugiperda* (Smith, 1797) (Lepidoptera: Noctuidae), is one of the most destructive agricultural pests, capable of damaging more than 300 plant species, especially those in the Poaceae, Asteraceae, and Fabaceae families [1]. It is one of the main pests affecting important agricultural commodities in the Americas [2,3,4] and has been found in Africa, Asia, and the Mediterranean region of Europe [5,6,7,8].

Control of *S. frugiperda* mainly relies on synthetic insecticides and genetically modified (GM) plants. However, because of this lepidopteran’s high fecundity and genetic plasticity, and the selection pressure from the intensive use of these tools, resistant populations have been frequently reported [9,10,11,12,13,14]. Among the synthetic insecticides used against this pest is chlorpyrifos, an organophosphate insecticide with acetylcholinesterase-inhibiting activity, commonly employed as a reference insecticide in toxicological bioassays with lepidopteran pests. In addition, inappropriate insecticide uses compromises natural-enemy populations and increases the risk of environmental contamination, highlighting the urgent need for more sustainable management alternatives [15,16].

The search for new molecules with lower environmental risk is becoming increasingly important, and in this context, plant essential oils (EOs) are being increasingly explored for pest control [16,17,18,19]. EOs are an abundant source of bioactive substances and generally have low persistence in soil, low environmental contamination, can be selective to natural enemies, and have different mechanisms of action that can delay the selection of resistant organism populations [20,21,22]. The secondary metabolites present in EOs can act as defense mechanisms against herbivorous insects [23,24,25]. As a result, EOs can cause acute toxicity, repellency and/or attraction, and paralysis of insect feeding and oviposition [23,26,27]. As a result of extensive research in this area, the commercialization of EO insecticides has increased significantly in North America and Europe in recent years, with market expansion in Latin America [15,28].

In this context, EOs have attracted increasing attention as promising alternatives for pest management. Those belonging to the Myrtaceae family [29] are gaining increasing interest in integrated pest management (IPM) programs, with emphasis on the species *Syzygium aromaticum* (L.) Merrill. & Perry, whose major compounds eugenol and (*E*)-caryophyllene are associated with ovicidal, larvicidal, and adulticidal effects; repellent activity; fumigant and contact toxicity [24,30]. Another relevant family is Apiaceae, which includes aromatic plants containing important EOs that can be exploited in different ways, such as in developing botanical insecticides [31,32,33]. The EOs of plants in this family have effective insecticidal action against a large number of agricultural insect species [31,34,35]. The species *Foeniculum vulgare* var. *dulce* (Mill.) Thell stands out because its major compound (*E*)-anethole and other chemical constituents are effective against insects, including aphicidal activity against the green peach aphid, *Myzus persicae* Sulzer (Hemiptera: Aphididae) [36], and larvicidal activity against the mosquito *Culex pipiens* Linnaeus (Diptera: Culicidae) [37], in addition to its broader potential as a botanical insecticide [34]. However, studies evaluating the potential of *S. aromaticum* and *F. vulgare* var. *dulce* EOs in controlling *S. frugiperda* are scarce.

Among the natural enemies of *S. frugiperda*, *Trichogramma pretiosum* Riley, 1879 (Hymenoptera: Trichogrammatidae) plays an important role in regulating pest populations under natural conditions and is the most widely marketed parasitoid for controlling fall armyworm in Brazil [38]. This species can reduce noctuid populations in corn crops by approximately 80%, resulting in productivity gains of up to 19.4% [39,40]. Furthermore, recent studies have highlighted the potential physiological selectivity of EOs and their major constituents for natural enemies, reinforcing their applicability in integrated pest management [19,41].

Given the challenges associated with synthetic insecticides and the need for selective, environmentally safe control strategies, this study hypothesizes that EOs of *S. aromaticum* and *F. vulgare* var. *dulce,* and their major compounds, are toxic to *S. frugiperda* and selective with respect to *T. pretiosum*. To this end, the objectives were: (i) to evaluate the acute toxicity of *S. aromaticum* and *F. vulgare* var. *dulce* EOs on *S. frugiperda*; (ii) to characterize the chemical composition of the EOs; (iii) to determine the toxicity of the major constituents; (iv) to investigate the effects of sublethal doses of EOs on biological parameters of *S. frugiperda*; and (v) to analyze the physiological selectivity of EOs and its major components for *T. pretiosum*.

## 2. Results

### 2.1. Chemical Characterization of EOs by GC and GC-MS

Chemical analysis of the EO of *S. aromaticum* indicated six constituents, with eugenol and (*E*)-caryophyllene as the major compounds. The EO of *F. vulgare* var. *dulce* contained eleven chemical compounds, with (*E*)-anethole being the most abundant (Table 1).

### 2.2. Acute Toxicity of EOs to S. frugiperda via Topical Application

**Figure 1:**
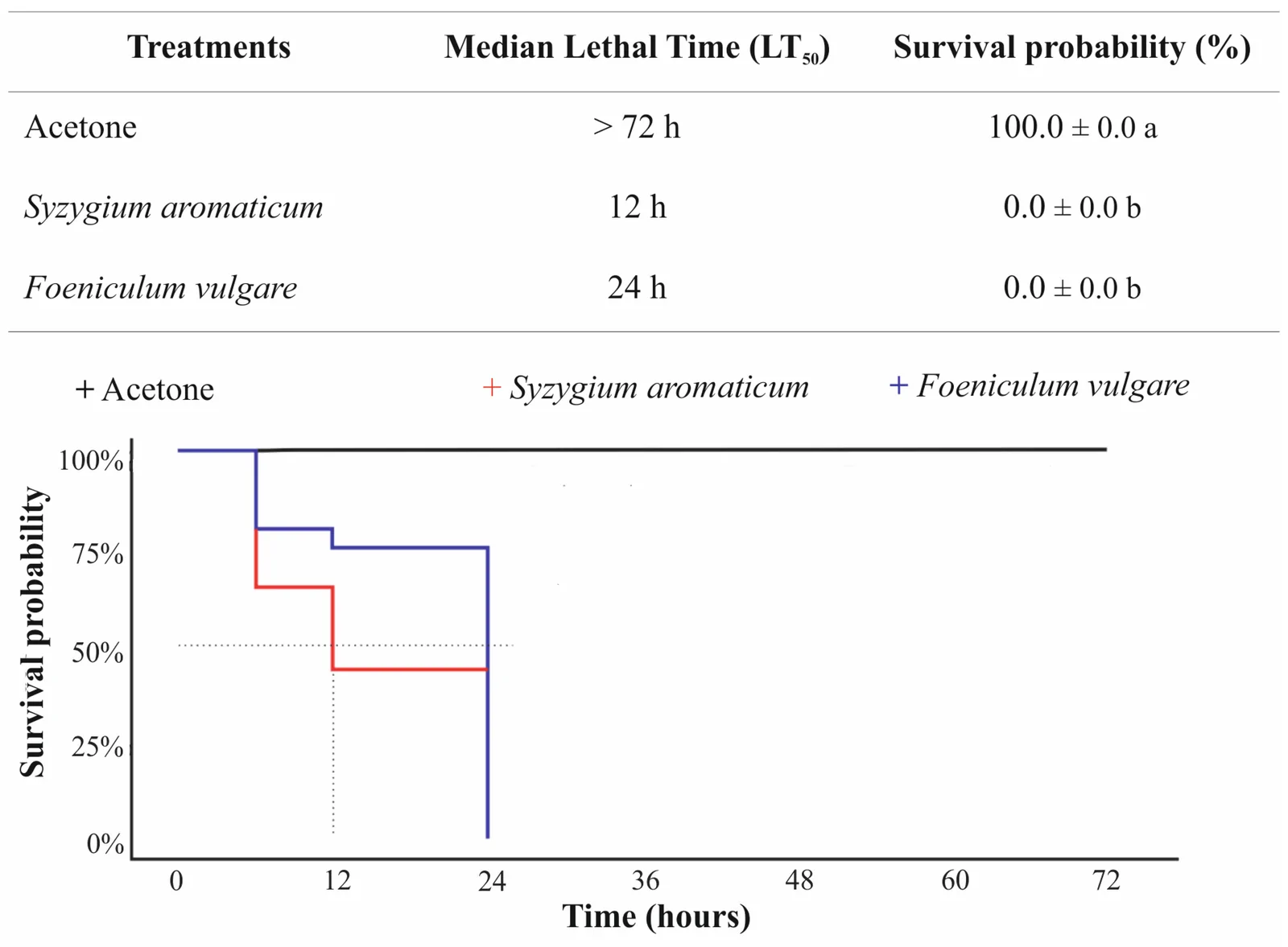
Survival analysis of *Spodoptera frugiperda* caterpillars treated with *S. aromaticum* and *Foeniculum vulgare* var. *dulce* essential oils via topical application. Means in the same column followed by different letters differ from each other (*p* < 0.05).

EOs from *S. aromaticum* and *F. vulgare* var. *dulce* caused 100% mortality of *S. frugiperda* caterpillars at a dose of 120 µg/caterpillar (χ2 = 268; d.f. = 5; *p* < 0.001). Caterpillars treated with *S. aromaticum* EO had a median lethal time (LT_50_) of 12 h, while treatment with *F. vulgare* var. *dulce* EO took twice as long to cause 50% mortality in the population (Figure 1).

### 2.3. Determination of Dose–Response and Time-Response Curves

After applying the treatments to second-instar caterpillars of *S. frugiperda*, it was found that the EO of *S. aromaticum* was 25% less toxic than that of *F. vulgare* var. *dulce*. The LD_50_ values of the EOs of *S. aromaticum* and *F. vulgare* var. *dulce* were, respectively, 1800- and 1360-fold higher than the LD_50_ estimated for chlorpyrifos (Table 2).

**Figure 2:**
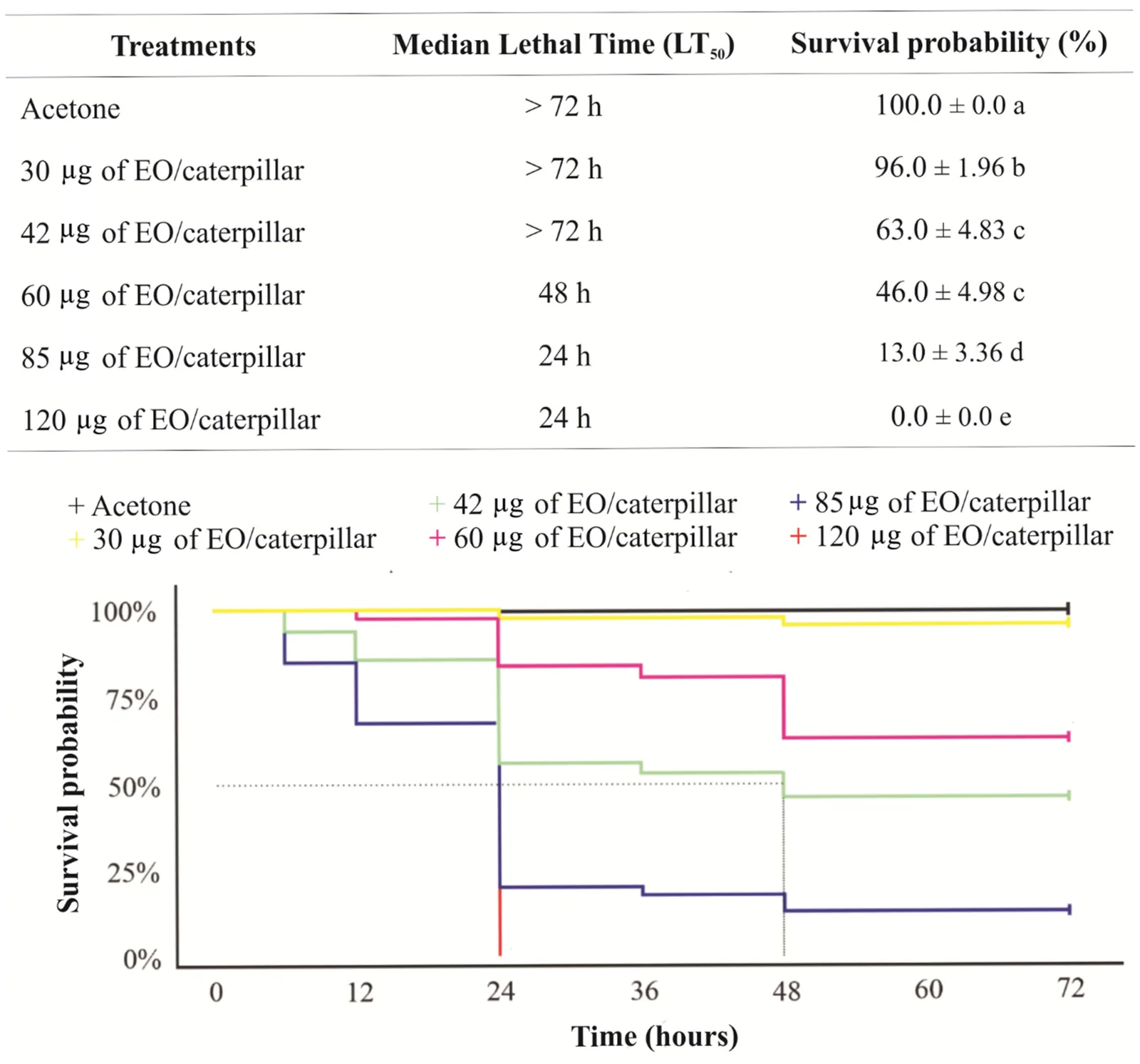
Survival analysis of *Spodoptera frugiperda* caterpillars treated with different doses of *Syzygium aromaticum* essential oil via topical application. Means in the same column followed by different letters differ from each other (*p* < 0.05).

Through survival analysis of *S. frugiperda* caterpillars that received different doses of *S. aromaticum* EO, differences were found between treatments (χ^2^ = 422; df = 5; *p* = <0.005). For doses of 85 µg of EO/caterpillar and 120 µg of EO/caterpillar, the LT_50_ was only 24 h (Figure 2).

**Figure 3:**
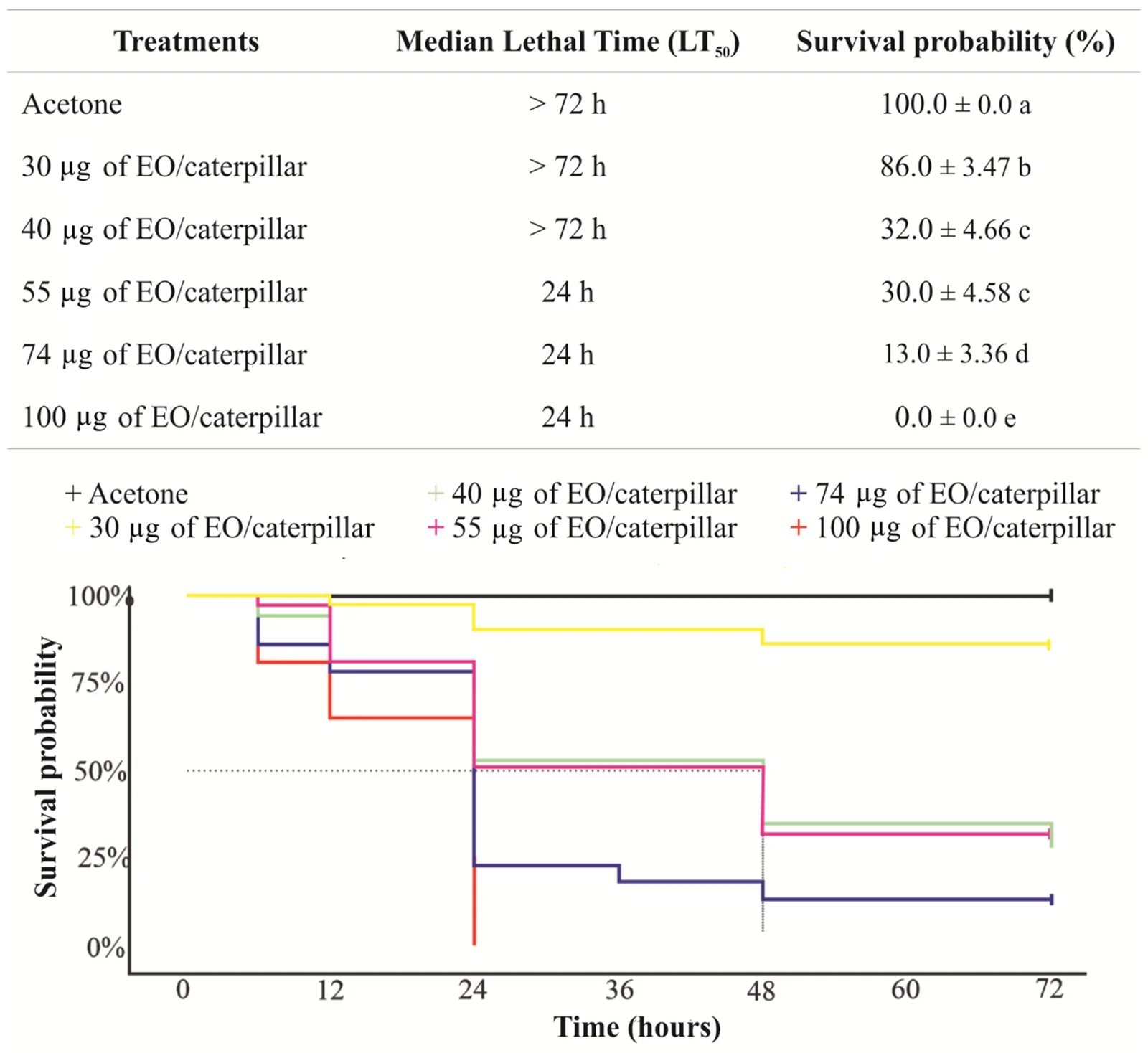
Survival analysis of *Spodoptera frugiperda* caterpillars treated with different doses of *Foeniculum vulgare* var. *dulce* essential oil via topical application. Means in the same column followed by different letters differ from each other (*p* < 0.05).

There was a reduction in the probability of survival of *S. frugiperda* caterpillars as the tested doses of *F. vulgare* var. *dulce* EO increased (χ^2^ = 355; df = 5; *p* = <0.005). For doses above 55 µg of EO/caterpillar, the LT_50_ was 24 h (Figure 3).

**Figure 4:**
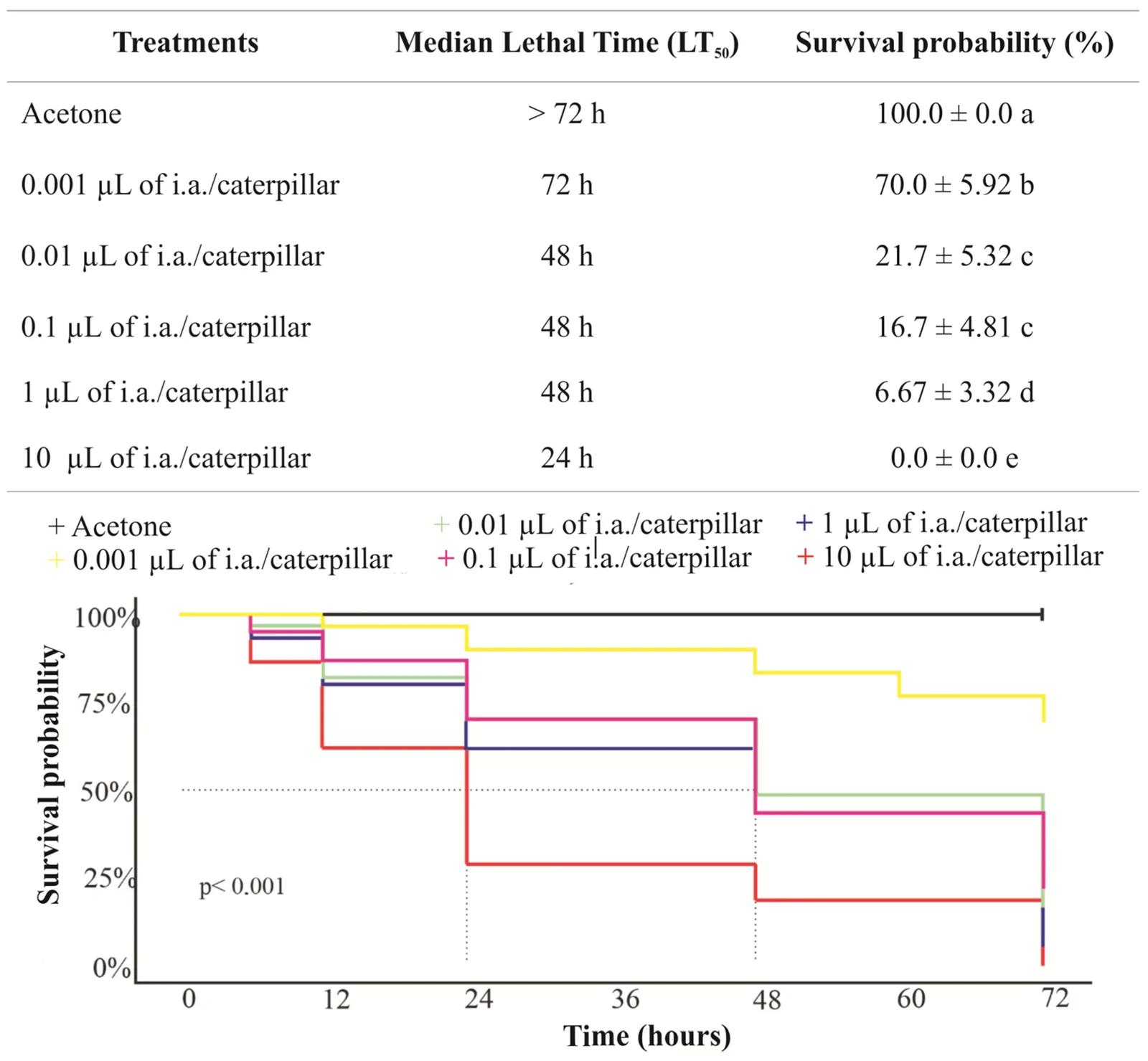
Survival analysis of *Spodoptera frugiperda* caterpillars treated with different doses of the active ingredient chlorpyrifos via topical application. Means in the same column followed by different letters differ from each other (*p* < 0.001).

The active ingredient chlorpyrifos was toxic to *S. frugiperda* caterpillars in proportion to the doses applied (χ^2^ = 202; df = 5; *p* < 0.001). At a dose of 10 µL of a.i./caterpillar, the LT_50_ was 24 h, while at doses of 0.01, 0.1, and 1 µL of a.i./caterpillar, the LT_50_ was 48 h. Survival probabilities were below 22% for doses greater than 0.01 µL of a.i./caterpillar (Figure 4).

### 2.4. Effects of the Major Compounds of EOs on S. frugiperda

**Figure 5:**
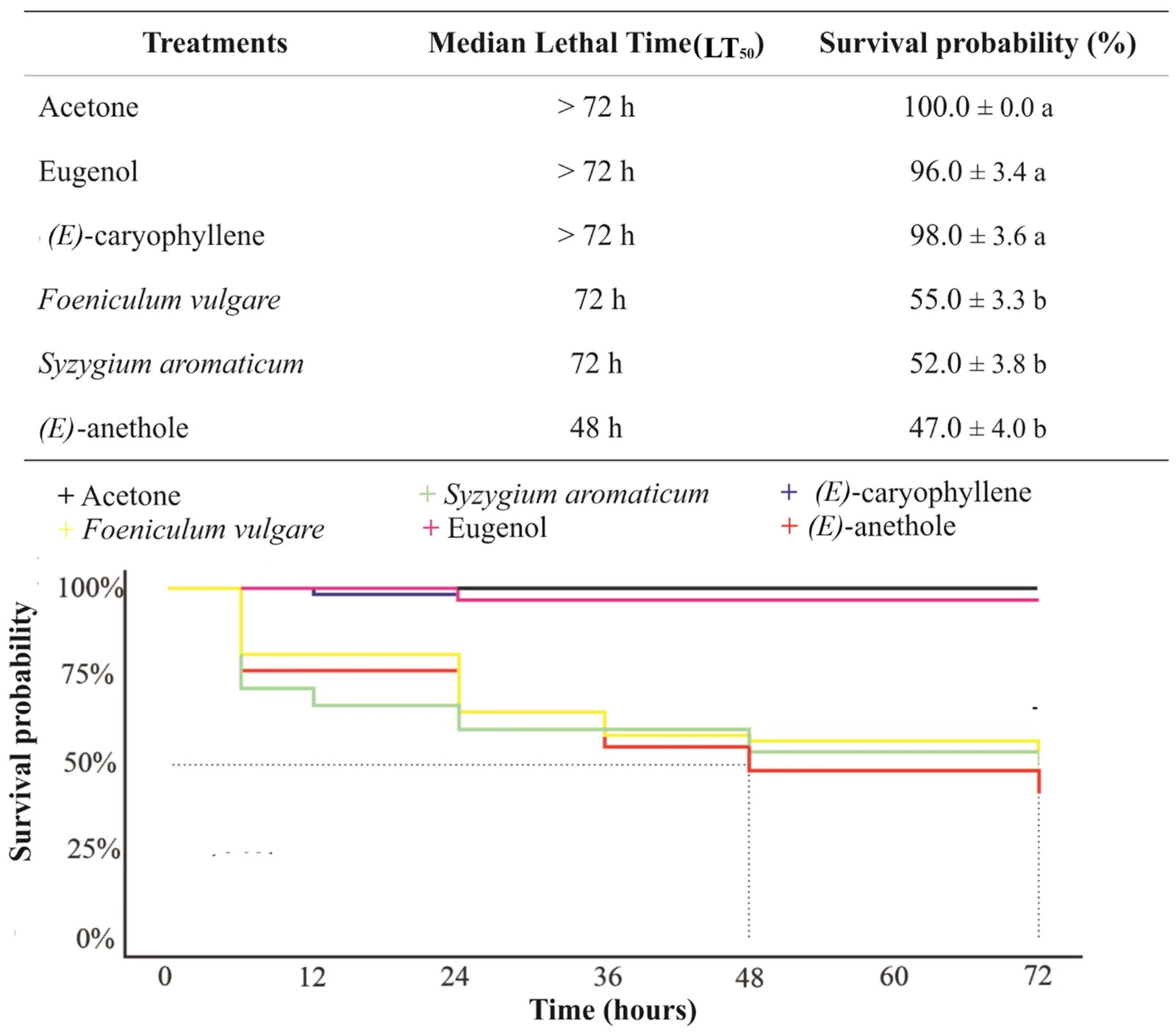
Survival analysis of *Spodoptera frugiperda* caterpillars treated with essential oils of *Foeniculum vulgare* var. *dulce* (LD_50_ 41.13 µg of EO/caterpillar) and *Syzygium aromaticum* (LD_50_ 54 µg of EO/caterpillar), and their major components, eugenol (41 µg of a.i./caterpillar), (*E*)-anethole (34 µg of a.i./caterpillar), and (*E*)-caryophyllene (10.75 µg of a.i./caterpillar), via topical application. Means in the same column followed by different letters differ from each other (*p* < 0.05).

Survival analysis showed differences between treatments (χ^2^ = 114; df = 5; *p* < 0.001). Eugenol and (*E*)-caryophyllene, tested individually, did not reproduce the insecticidal activity of *S. aromaticum* EO. It was found that (*E*)-anethole accounts for most of the insecticidal activity of *F. vulgare* var. *dulce* EO (Figure 5).

### 2.5. Effects of Sublethal Doses of EOs on the Life History Parameters of S. frugiperda

EOs increased the duration of the egg–larva period, with the highest average in the *F. vulgare* var. *dulce* EO treatment. Regarding the egg-to-pupa period, *S. aromaticum* EO and chlorpyrifos caused the greatest reduction. EO treatments reduced adult longevity. Differences in female and male life cycles were observed between treatments, with reduced life cycles under *S. aromaticum* EO and an increased life cycle in males under *F. vulgare* var. *dulce* EO (Table 3).

**Figure 6:**
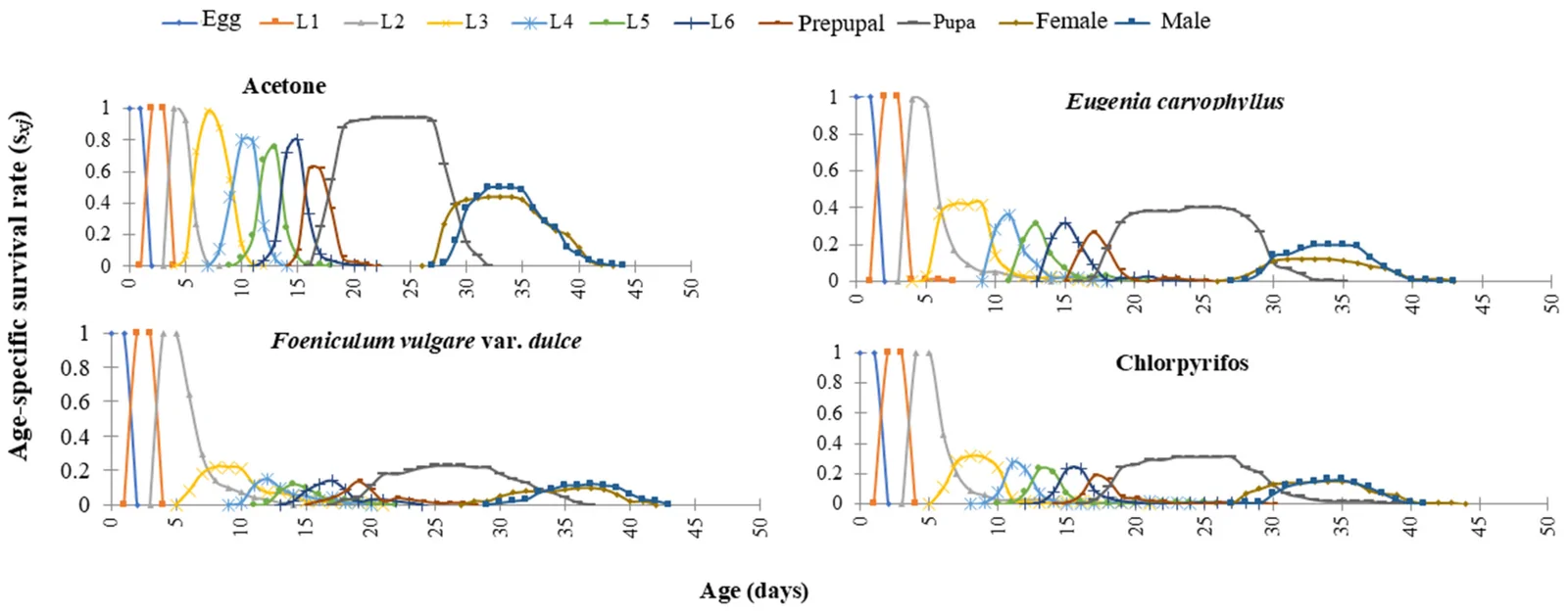
Age-stage-specific survival rate (S_xj_) of *Spodoptera frugiperda* treated with the LD_50_ of *Syzygium aromaticum* essential oil (LD_50_ 54 µg of EO/caterpillar), *Foeniculum vulgare* var. *dulce* essential oil (LD_50_ 41.13 µg of EO/caterpillar), chlorpyrifos, and acetone.

The age-specific survival rate (S_xj_) was negatively affected by the treatments. Both OEs prolonged the pupal stage and presented lower S_xj_ values from the third instar onwards (Figure 6).

**Figure 7:**
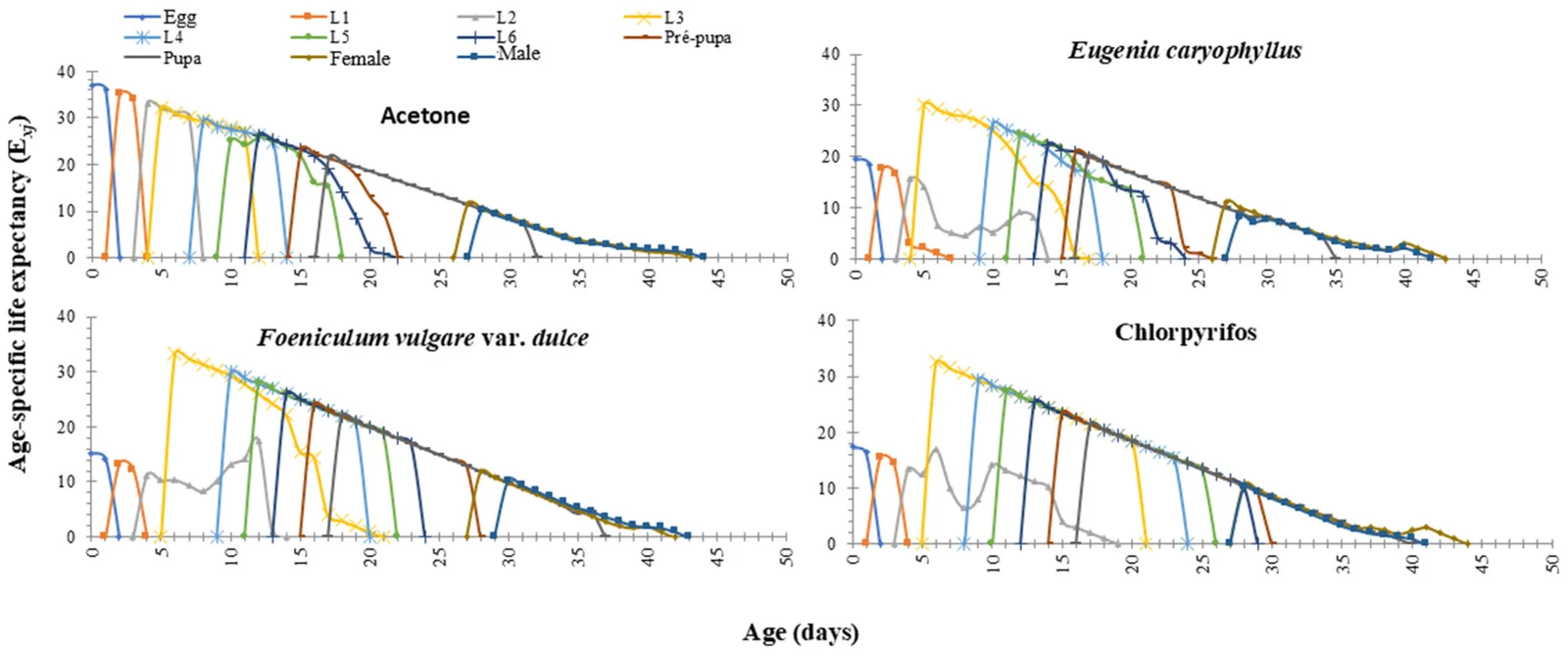
Age-stage-specific life expectancy (E_xj_) of *Spodoptera frugiperda* treated with the LD_50_ of *Syzygium aromaticum* essential oil (LD_50_ 54 µg of EO/caterpillar), *Foeniculum vulgare* var. *dulce* essential oil (LD_50_ 41.13 µg of EO/caterpillar), chlorpyrifos, and acetone.

It was found that age-specific life expectancy (E_xj_) decreased with age in the acetone treatment, while in the other treatments there was a peak in the third instar and a subsequent decrease throughout the life cycle (Figure 7).

**Figure 8:**
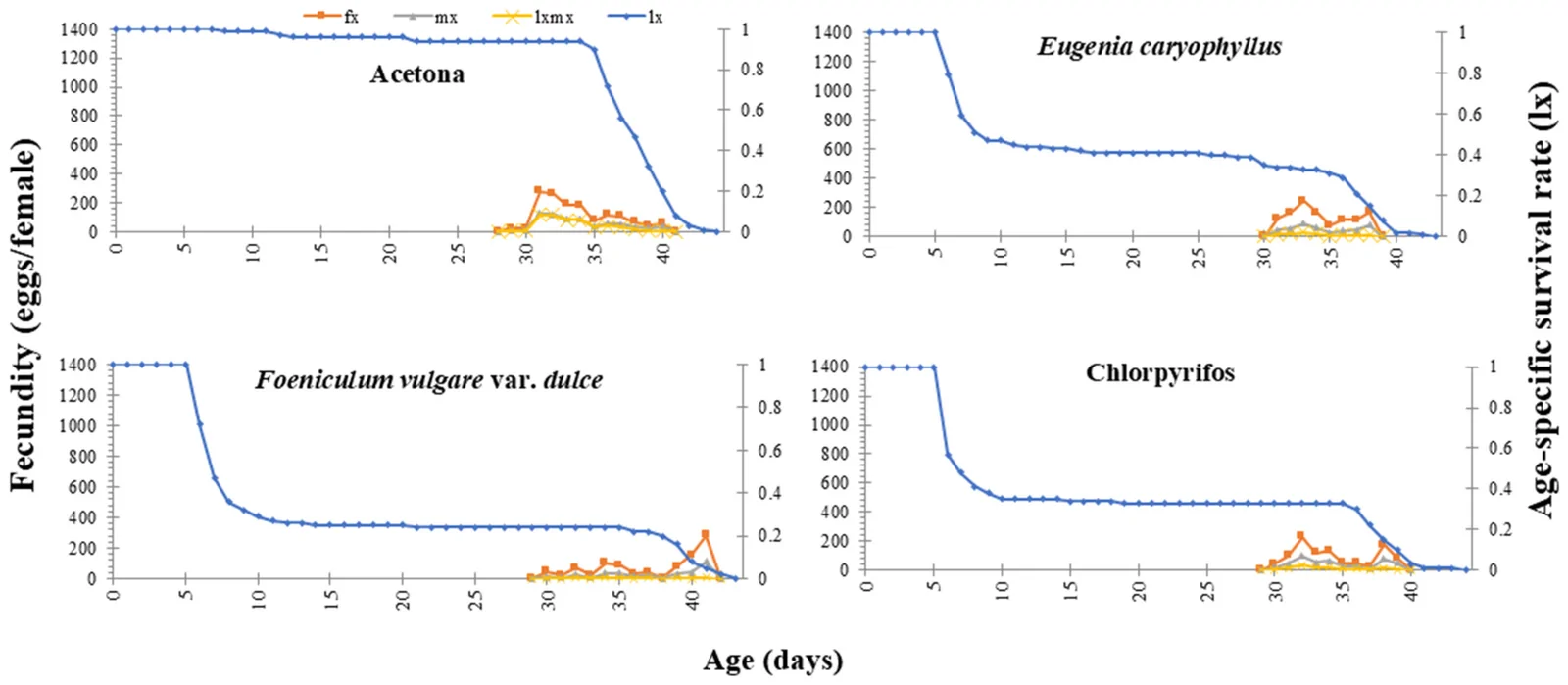
Age-specific survival rate (l_x_), age-stage-specific fecundity (f_xj_), age-specific fecundity (m_x_), and age-specific maternity (l_x_m_x_) of *Spodoptera frugiperda* treated with the LD_50_ of *Syzygium aromaticum* essential oil (LD_50_ 54 µg of EO/caterpillar), *Foeniculum vulgare* var. *dulce* essential oil (LD_50_ 41.13 µg of EO/caterpillar), chlorpyrifos, and acetone.

It was found that the age-specific survival rate curve (l_x_) decreased abruptly around the 5th day of life of the insects in all treatments, except in the acetone treatment, where the reduction was observed only on the 35th day of the life cycle. There was a delay in age-specific fertility (f_xj_) in the first oviposition in the treatment with EO from *S. aromaticum*. The peak oviposition occurred at the end of the cycle in the treatment with EO from *F. vulgare* var. *dulce*, which also prolonged the reproductive period (l_x_m_x_) (Figure 8).

Total and effective fecundity were negatively affected by the EOs, with *F. vulgare* var. *dulce* EO causing the greatest reduction in these biological parameters. The EO of *F. vulgare* var. *dulce* also reduced the adult pre-oviposition period (APOP) and increased the total pre-oviposition period (TPOP). The lowest maximum daily fecundity (MDF) was observed in the treatments with EOs (Table 4).

The number of replicates used to estimate the reproductive parameters (Table 4) reflect the cumulative mortality of treated insects from the larval stage through adult emergence and pairing; this reduction was most pronounced in the *F. vulgare* treatment, consistent with its stronger sublethal effects on larval and pupal development (Table 3).

**Figure 9:**
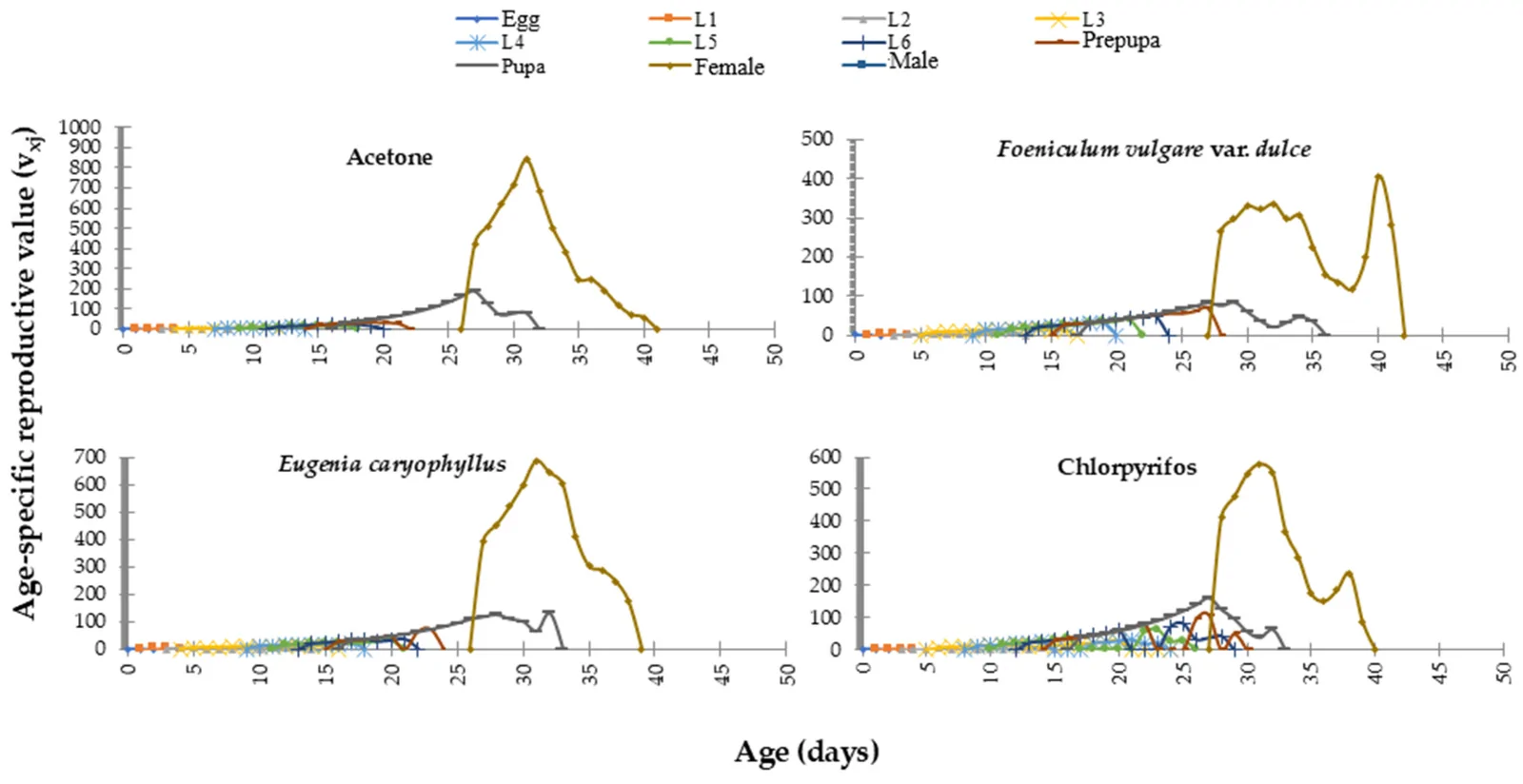
Age-specific reproductive value (v_xj_) of *Spodoptera frugiperda* in treatments with acetone, *Foeniculum vulgare* var. *dulce*, *Eugenia caryophyllus*, and chlorpyrifos. L_1_ = 1st instar caterpillar, L_2_ = 2nd instar caterpillar, L_3_ = 3rd instar caterpillar, L_4_ = 4th instar caterpillar, L_5_ = 5th instar caterpillar, and L^6^ = 6th instar caterpillar.

In the *S. aromaticum* and chlorpyrifos treatments, females reached their age-specific reproductive peak (v_xj_) on the 31st day of the life cycle, with v_xj_ values of 842; 688, and 579 eggs, respectively. In the no-treatment group with *F. vulgare* var. *dulce*, the reproductive peak occurred on the 40th day of the cycle, presenting a v_xj_ value of 407 eggs (Figure 9).

Insects treated with the lower essential oils showed lower intrinsic growth rate (r), finite growth rate (λ), and net reproductive rate (R0) values. Only the oil of *F. vulgare* var. *dulce* inhibited the gross reproductive rate (GRR) compared to insects treated only with acetone (Table 5).

### 2.6. Selectivity of EOs and (E)-Anethole for Adult Females of T. pretiosum

The longevity of *T. pretiosum* females that came into contact with *E. kuehniella* eggs that received the treatments was affected (χ2 = 9.22; df = 2; *p* < 0.001). The major compound, (*E*)-anethole, reduced insect longevity more than the acetone-only treatment. In the EO of *S. aromaticum*, females parasitized fewer eggs compared to the other treatments (F = 189.47; df = 4; *p* < 0.001). Treatments also affected emergence percentage (F = 15.9; df = 2; *p* < 0.001), with lower averages for the *F. vulgare* var. *dulce* and (*E*)-anethole treatments than for acetone. Females that came into contact with host eggs treated with chlorpyrifos and *S. aromaticum* showed a longevity of 1 day; these treatments resulted in low parasitism and no emergence of the F_1_ generation. Because these two treatments showed no variability, they were not included in the statistical analysis. Regarding the classification of compounds based on emergence reduction (IOBC) [42,43], *F. vulgare* var. *dulce* and (*E*)-anethole were selected as moderately harmful (class 3), and the *S. aromaticum* and chlorpyrifos treatments were harmful (class 4) (Table 6).

### 2.7. Selectivity of EOs and (E)-Anethole for T. pretiosum in Its Immature Stage

Differences occurred between treatments when *E. kuehniella* eggs containing parasitoids in the egg–larva period (F = 27.916; df = 4; *p* < 0.001), in the pre-pupal stage (F = 38.974; df = 4; *p* < 0.001) and in the pupal stage (F = 32.564; df = 4; *p* < 0.001) were treated, with the EO of *S. aromaticum* being classified as moderately harmful (class 3), and the major compound of the EO of *F. vulgare* var. *dulce*, (*E*)-Anethole, being harmless (class 1) (Table 7).

## 3. Discussion

This study demonstrated that the EOs of *F. vulgare* var. *dulce* and *S. aromaticum* caused high mortality (LD_50_ = 41 and 54.2 µg/caterpillar, respectively) against *S. frugiperda* following topical application. Similar bioactivity has been reported for other *Spodoptera* species, supporting the broad-spectrum insecticidal potential of these botanical products. *Foeniculum vulgare* EO has previously induced significant mortality in *S. frugiperda* caterpillars [44], while ingestion assays showed strong antifeedant activity against *Spodoptera exigua* (Hübner, 1808) and *Spodoptera litura* (Fabricius, 1775) (Lepidoptera: Noctuidae) [45,46]. These findings reinforce the potential of *F. vulgare* var. *dulce* EO as a promising botanical insecticide against economically important noctuid pests. Likewise, although relatively few studies have investigated the activity of *S. aromaticum* EO against *Spodoptera* spp., previous reports have attributed its insecticidal and antifeedant properties mainly to its high eugenol content, suggesting that this constituent plays a major role in its biological activity.

The chemical composition of the EOs evaluated in this study is within the range reported by other authors, with eugenol (76.8–78%) and (*E*)-caryophyllene (6–17%) in the EO of *Eugenia caryophyllus* (*S. aromaticum)* [24,47,48,49], and (*E*)-anethole (68.3–77.7%) present in the EO of *F. vulgare* [37,50,51]. However, several factors can affect the chemical composition of EOs, such as climatic and geographical conditions [52,53]; abiotic factors such as salinity [54], altitude, and soil type [55]; growth stage of the plants collected [56], extraction method [57], different plant varieties and/or parts of the plant that will be used for EO extraction [58]. Such variability may directly influence insecticidal efficacy and should therefore be considered when comparing biological activity among studies.

In the dose–response bioassay, the estimated LD_50_ values for *S. aromaticum and F. vulgare* var. *dulce* EO were 54.2 µg EO/caterpillar and 41 µg EO/caterpillar for *S. frugiperda*. The low LD_50_ value for the insecticide chlorpyrifos (0.03 µg a.i./caterpillar) suggests that the population evaluated remains susceptible to this insecticide under the laboratory conditions tested. However, together with their effects on demographic parameters, reducing the intrinsic growth rate (r) and net reproductive rate (*R*0) relative to the acetone control (Table 5), demonstrate their potential as botanical alternatives for integrated pest management. In addition to causing acute mortality, these EOs reduced population growth, indicating that their overall impact extends beyond direct lethal effects.

When comparing the LD_50_ values found for other EOs considered promising for the control of this lepidopteran pest, it can be observed that the estimated LD_50_ values for EOs *S. aromaticum* and *F. vulgare* var. *dulce*, in the present study, are lower than those estimated for the EO of *Ocimum basilicum* L., 1753 (Lamiaceae), where LD_50_ = 480.00 (447.51–512.48 μg/caterpillar) [59] and for the EO of *Piper hispidinervum* C. DC. (Piperaceae) with LD_50_ = 264.5 (207.40–324.50 μg/caterpillar) [60]. These comparisons further support the high insecticidal potential of the investigated EOs and highlight their suitability for further development as environmentally compatible tools for managing *S. frugiperda*.

In the present study, the tested doses of the major compounds of *S. aromaticum* EO (LD_50_ = 41.13 µg/caterpillar for eugenol and LD_50_ = 10.75 µg/caterpillar for (*E*)-caryophyllene) did not cause mortality in *S. frugiperda* when tested individually at doses equivalent to their proportions in the EO LD_50_. In contrast, the complete EO caused substantially higher mortality than either isolated compound at equivalent doses (Figure 5), suggesting that its bioactivity cannot be attributed solely to its major constituents. Similar observations have been reported for other essential oils, whose biological activity results from complex interactions among major and minor constituents rather than from a single dominant compound [61,62]. Indeed, synergistic interactions may enhance insecticidal efficacy by increasing cuticular penetration, improving compound stability, or targeting multiple physiological pathways simultaneously, whereas antagonistic interactions may also modulate toxicity [35].

Although previous studies have attributed the insecticidal activity of *S. aromaticum* EO mainly to eugenol, eugenol acetate, and *β*-caryophyllene [63,64], the present findings indicate that these compounds alone are insufficient to reproduce the biological activity of the whole EO against *S. frugiperda*. Likewise, Hummelbrunner and Isman [65] reported that eugenol exhibited insecticidal and antifeedant activity against *S. litura* (LD_50_ = 65.5 µg/caterpillar), indicating that the biological response to this compound may vary among *Spodoptera* species. Therefore, the insecticidal activity observed in the present study likely reflects the combined action of multiple constituents, highlighting the importance of preserving the EO’s natural chemical complexity rather than focusing exclusively on isolated compounds.

In the present study, the *(E)*-anethole was used in a concentration proportional to its concentration in the activity of *F. vulgare* var. *dulce* EO, as determined by GC-MS analysis. Thus, the insecticidal activity of this EO is possibly due to the action of its major constituent. The insecticidal effect of (*E*)-anethole on *Spodoptera littoralis* (Boisduval, 1833) (Lepidoptera: Noctuidae) was found in the work of [66], where an LD_50_ of 18 μg/caterpillar with acute toxicity was verified. The effect of (*E*)-anethole was also evaluated for *S. litura* caterpillars by Hummelbrunner and Isman [65], who found an LD_50_ of 157.6 μg/caterpillar and observed an anti-feeding effect.

The mechanisms of (*E*)-anethole on arthropod pests are not yet fully understood. Ref. [67] evaluated how *Myzus persicae* (Sulzer, 1776) (Hemiptera: Aphididae) responded at the molecular level to the activity of (*E*)-anethole and found a knockdown effect on an ABC transporter gene (ABCG4) and a DnaJ gene (DnaJC1), which resulted in an increased mortality rate, indicating the involvement of these two genes in the susceptibility of this aphid to (*E*)-anethole. In addition, fumigation assays with *Cryptolestes ferrugineus* (Stephens, 1831) (Coleoptera: Laemophloeidae) and *Tribolium castaneum* (Herbst, 1797) (Coleoptera: Tenebrionidae) demonstrated that (*E*)-anethole inhibits acetylcholinesterase (AChE) activity, impairing neurotransmission in these beetles [68,69]. Although similar molecular studies have not yet been conducted for *S. frugiperda*, these findings provide evidence that (*E*)-anethole interferes with essential physiological processes in insects and may act through conserved neurotoxic mechanisms.

The EOs or their major constituents can act at different sites in the insect nervous system, such as AChE, octopamine receptors, and GABA-controlled chloride channels [70,71]. Eugenol has been shown to inhibit AChE [72,73]. AChE is an enzyme essential for the hydrolysis of acetylcholine located in nerve cells and at the neuromuscular junction [74]. High rates of AChE inhibition prevent interruption of nerve impulse propagation; thus, the nervous system undergoes successive, uncontrolled stimulation and cellular hyperactivity, leading to insect death [75]. Thus, the mode of action of some plant EOs or their major constituents is similar to that of organophosphate (chlorpyrifos) and carbamate insecticides, which are commonly used to control *S. frugiperda* [76,77,78].

In the present study, (*E*)-anethole was considered the major compound of the EO of *F. vulgare* var. *dulce* and caused a reduction in fertility (Table 4) and in the demographic parameters of *S. frugiperda* (Table 3) such as intrinsic growth rate (r), finite growth rate (λ), net reproduction rate (R_0_), and gross reproduction rate (GRR). These findings show that, beyond its acute insecticidal activity, (*E*)-anethole reduces reproductive output and demographic performance (Table 3 and Table 4), suppressing population growth. Such effects are particularly relevant for integrated pest management because reduced reproductive performance may limit pest population expansion over successive generations, even when complete mortality is not achieved.

The present findings are similar to other studies, such as that of [76,77], where the compound (*E*)-anethole reduced the reproduction of *S. frugiperda* with adverse effects on its gonads. The caterpillars showed nutrient deficiency, with reduced fertility and testicular apoptosis in the adults. Many EO components interfere with reproduction, reducing insect fertility and fecundity, especially eugenol and (*E*)-anethole [79,80]. Therefore, the reductions observed in the demographic parameters in the present study likely reflect cumulative physiological impairments affecting both survival and reproductive performance.

Sublethal effects are increasingly recognized as important indicators of insecticide efficacy because they directly influence population dynamics and long-term pest suppression. Consequently, evaluating demographic parameters through life table analyses provides a more realistic assessment of the ecological impact of botanical insecticides than mortality alone. Moreover, hormetic responses following exposure to low or sublethal doses of bioactive compounds reinforce the importance of investigating both lethal and sublethal effects when assessing the potential of plant-derived insecticides for pest management [81,82].

With regard to the effects observed in this study on *T. pretiosum*, the reduction in parasitism in *E. kuehniella* eggs may be due to the repellent effect caused by the combined action of the chemical constituents present in the EOs, corroborating the information provided by [83]. The immature stage of parasitoids is generally more protected within the host [84]; however, EOs can penetrate the egg and reach the parasitoids in the young stage and cause mortality [85,86,87], as observed in the present study for the EO of *S. aromaticum* on *T. pretiosum*.

The selectivity of these EOs to *T. pretiosum* was dependent on the developmental stage exposed: adult females exposed by direct contact with treated eggs were more strongly affected (classes 3 and 4, Table 6) than the immature stages protected within the host egg (classes 1 and 2, Table 7), reflecting the reduced physical exposure of eggs, larvae, and pupae developing inside the chorion. Despite these effects, *F. vulgare* var. *dulce* EO and its major constituent (*E*)-anethole exhibited low toxicity to immature parasitoids.

Despite these effects, *F. vulgare* var. *dulce* EO and its major constituent (*E*)-anethole exhibited low toxicity to immature parasitoids during specific developmental stages, indicating a greater degree of selectivity than *S. aromaticum* EO. This selectivity suggests that these botanical compounds may be compatible with biological control programs based on *T. pretiosum*, although additional semi-field and field studies are necessary to confirm their safety under practical application conditions and to evaluate their integration into integrated pest management programs.

This study has some limitations that should be acknowledged. First, bioassays were conducted exclusively under controlled laboratory conditions, and the persistence and efficacy of the EOs under field conditions remain to be evaluated. Second, the reduced number of females available for reproductive assessment in the *F. vulgare* var. *dulce* treatment, resulting from cumulative sublethal mortality, limits the statistical power of comparisons involving this treatment’s reproductive parameters. Third, the major compounds were tested in isolation, without combination assays that would allow synergistic or antagonistic interactions among EO constituents to be quantified. Finally, the biochemical or molecular mode of action of these EOs and their major compounds was not investigated in *S. frugiperda*, and this remains an important direction for future research.

## 4. Materials and Methods

### 4.1. Rearing of S. frugiperda and T. pretiosum

*Spodoptera frugiperda* caterpillars were reared as described by [19]. *Trichogramma pretiosum* was multiplied using eggs from the alternative host *E. kuehniella*. The adults were fed pure honey and kept in an air-conditioned room at 25 ± 2 °C, 60 ± 10% RH, and a 14-h photophase, as described by [88].

### 4.2. EOs and Chemical Characterization

The EOs of *S. aromaticum* (plant stems) and *F. vulgare* var. *dulce* (seeds) were purchased from the company Ferquima Indústria e Comércio Ltda., Vargem Grande Paulista, São Paulo-Brazil (lots 192 and 215, respectively; https://www.ferquima.com.br). Both were obtained by steam distillation in a Clevenger apparatus. They were analyzed by gas chromatography–mass spectrometry (GC–MS) using a gas chromatograph coupled to a mass spectrometer (model QP2010, Shimadzu, Kyoto, Japan), equipped with a capillary column RTX-5MS (30 m × 0.25 mm ID × 0.25 μm film thickness; Restek, Bellefonte, PA, USA). The EOs were diluted in acetone to a concentration of 10 mg/mL and 1 μL of each solution was injected in split mode (1:20 ratio) into injector at 220 °C. The analysis conditions followed the recommendations of Adams [87]: helium at 1.0 mL/min was used as carrier gas; initial column temperature, 60 °C; column temperature rise rate, 2 °C/min up to 200 °C, where after 200 °C, the rise rate increased to 5 °C/min; final column temperature, 250 °C; temperature of the interface between the chromatograph and the mass spectrometer 220 °C; ionization of molecules in the spectrometer through electron impact at 70 eV; mass/charge (*m*/*z*) range analyzed in mass spectrometer, 45–400; mass spectrum acquisition time, 0.5 s. Components were identified by comparing their mass spectra to those in the NIST 05 Mass Spectral Library 2005 using the equipment’s onboard software. Any substances with less than a 90% similarity match are considered unidentified. Retention indices (RI) of the compounds were calculated by comparing their retention times with those of solutions containing linear alkanes (C_9_-C_20_), according to the following formula: RI = {100Pz + 100 [(RT − RTPz)/(RTPz + 1 − RTPz)]}, where Pz = number of carbon atoms of the linear alkane with a retention time immediately lower than that of the compound to be identified in the chromatogram; RT = retention time (min) of the substance to be identified in the chromatogram; RTPz = retention time (min) of the linear alkane with a number of carbon atoms equal to Pz, and RTPz + 1 = retention time (min) of the linear alkane with a number of carbon atoms equal to Pz + 1. Substances with calculated RI values corresponding to an error ≥ 3% in relation to the RI described by Adams [89] were considered unidentified.

### 4.3. Bioassays with S. frugiperda

#### 4.3.1. Acute Toxicity of Essential Oils in a Topical Application Assay

The EOs of *S. aromaticum* and *F. vulgare* var. *dulce* were previously diluted in acetone to 120 µg and applied topically to the back of 72-h-old *S. frugiperda* caterpillars measuring 0.4 mm in length, previously fed an artificial diet. Each caterpillar received 1 µL of the solution on its back with the aid of a microsyringe (Hamilton^®^, Reno, NV, USA, 25 μL). The negative control was acetone, the solvent used to dilute the EOs. The design was completely randomized, with 60 replicates per treatment, each consisting of a caterpillar placed in a glass tube (8 cm × 2.5 cm) containing a piece of artificial diet (1.5 cm × 1.5 cm). Assessments of the number of dead insects were performed at 6, 12, 24, 36, 48, and 72 h after topical treatment. The test was performed twice on different days.

#### 4.3.2. Determination of Dose–Response and Time-Response Curves

The EOs were separately dissolved in acetone to obtain working solutions at concentrations corresponding to the target doses. The doses used were determined by arithmetic progression in previous tests, aiming to obtain mortality values ranging from 20 to 90% [90]. The EO of *S. aromaticum* was applied to caterpillars at doses of 30, 42, 60, 85, and 120 µg of EO/caterpillar, while the EO of *F. vulgare* var. *dulce* was used at doses of 30, 40, 55, 74, and 100 µg of EO/caterpillar. To compare the bioactivity of the EOs with that of a commercial insecticide, the active ingredient (a.i.) chlorpyrifos (95% purity, Sigma-Aldrich^®^, Burlington, MA, USA) was used. The active ingredient was used at doses of 0.001, 0.01, 0.1, 1, and 10 µg of a.i./caterpillar and was previously solubilized in acetone. Each 72-h-old *S. frugiperda* caterpillar received 1 µL of the solution, as described above (Section 4.3.1). The negative control consisted of acetone alone. The design was completely randomized with 100 replicates per treatment. The number of live insects was assessed at 6, 12, 24, 36, 48, and 72 h after application of the treatments to calculate the median lethal time (LT_50_). To calculate the lethal dose 50 (LD_50_), insect mortality was assessed 48 h after treatment application.

#### 4.3.3. Effects of the Major Compounds in EOs

The effects of the major compounds eugenol and (*E*)-caryophyllene present in the EO of *S. aromaticum* and (*E*)-anethole in the EO of *F. vulgare* var. *dulce* were evaluated on *S. frugiperda*. The dose of each compound was determined by the following formula: D = LD_50_ × (T/100), where D = dose of the pure compound; LD_50_ = lethal dose of EO to kill 50% of the population (54 µg/caterpillar for *S. aromaticum* and 41 µg/caterpillar for *F. vulgare*) (Table 2), and T = content of the compound present in the EO (Section 2.1).

The treatments used consisted of EO from *S. aromaticum* (LD_50_ = 54 µL of EO/caterpillar), and its major components eugenol (41.13 µg of the component/caterpillar) and (*E*)-caryophyllene (10.75 µg/caterpillar); EO of *F. vulgare* var. *dulce* (LD_50_ = 41 µg of EO/caterpillar) and the major compound: (*E*)-anethole (34 µg/caterpillar) (Section 4.3.1). The negative control consisted of the solvent acetone, since it was used to dilute the treatments. Each *S. frugiperda* caterpillar received 1 µL of the solution, as described above (Section 4.3.1). The design used was completely randomized with 60 replicates for each treatment, as described above (Section 4.3.1). Insect mortality was assessed at 6, 12, 24, 36, 48, and 72 h after application of the treatments.

#### 4.3.4. Table of Life History and Demographic Parameters of the *S. frugiperda* Caterpillar

The effects of sublethal doses (LD_50_) of *S. aromaticum* EO (LD_50_ = 54 µg of EO/caterpillar) and *F. vulgare* var. *dulce* (LD_50_ = 41 µg of EO/caterpillar), the insecticide chlorpyrifos (positive control) (LD_50_ = 0.03 µg of a.i./caterpillar), and acetone (negative control) were evaluated on the life history parameters of *S. frugiperda*. Each 72-h-old *S. frugiperda* caterpillar received 1 µL of the solution, as described above (Section 4.3.1). The negative control consisted of acetone only. The design was completely randomized with 100 replicates per treatment. Every 3 days, a new piece of diet (1.5 cm × 1.5 cm), without treatment, was offered to the caterpillars until they reached the pupal stage. Because treatments were applied at the LD_50_, approximately half of the individuals died within 48 h of application; additional losses occurred during subsequent larval development and the pupal stage as a result of sublethal effects, further reducing the number of insects that reached adulthood and could be paired for reproductive assessment.

Survival, instar duration, and egg–larva and pupa development time of the insects were evaluated daily after application of the EOs. To evaluate the effects of OEs on adults from treated caterpillars, pairs with a maximum age difference of 48 h were formed and placed individually in cylindrical PVC cages (10 cm high × 10 cm in diameter). The cages were closed at the top with voile fabric and lined internally with sulfite paper, which served as a substrate for oviposition. The adults were fed a 10% aqueous honey solution. The eggs were collected and kept in 10 cm diameter Petri dishes for fertility assessment. The oviposition period, fertility, survival, and longevity of males and females (F_0_) were also evaluated.

### 4.4. Bioassays with T. pretiosum

#### 4.4.1. General Procedures

The insects used in the bioassays were obtained from a population of approximately 100,000 specimens of *T. pretiosum* from Koppert Biological Systems, Charqueada, São Paulo, Brazil, and kept in eggs of *E. kuehniella* at the Laboratory of Ecotoxicology and IPM (LEMIP) at the Federal University of Lavras, Lavras, Minas Gerais, Brazil. The treatments consisted of *S. aromaticum* EO (92.6 µg of EO/caterpillar) and *F. vulgare* var. *dulce* (70 µg of EO/caterpillar); the major component *(E)*-Anethole (58 µg of the compound/caterpillar); the active ingredient chlorpyrifos (0.3 µL of a.i./caterpillar) (positive control); and the negative control, acetone. The doses used in the treatments corresponded to the LD_90_ values found for *S. frugiperda* (Section 2.3). The dose of the major component was determined by the following formula: D = LD_90_ × (T/100), where D = dose of the pure compound; LD_90_ = lethal dose of essential oil for 90% of the population (Section 2.3), and T = content of the compound present in the EO (Section 2.1). The treatments were solubilized in acetone, which was therefore used as a negative control.

#### 4.4.2. Selectivity of OEs and (*E*)-Anethole for Adult Females

Female *T. pretiosum* up to 12 h old were placed individually in glass tubes (8 cm high × 2.5 cm in diameter), each containing one blue cardboard card (5 cm long × 0.5 cm wide) with approximately 125 *E. kuehniella* eggs up to 24-h-old. Before being attached to the cards using gum arabic diluted to 50% in water, the eggs were rendered unviable under a germicidal lamp for 45 min. The cards containing the *E. kuehniella* eggs were immersed in the treatment solutions, as mentioned in Section 4.4.1 for each EO, for 5 s [91] and kept at room temperature to remove excess solution from the egg surfaces. Each treated card was then placed in a glass tube containing one female parasitoid. The insects were fed using a drop of pure honey added to the wall of each tube.

The experiment was conducted in a completely randomized with 30 biological replicates per treatment, with each replicate consisting of one individually maintained female parasitoid exposed to one treated card containing approximately 125 host eggs. The individual female was considered the experimental unit, whereas the eggs on each card were considered subsamples and were not treated as independent replicates. After 24 h of exposure to the treated eggs, the females were removed and placed individually in new tubes for longevity assessment. The cards containing the exposed eggs were kept in the tubes to assess the numbers of parasitized eggs and emerged insects.

#### 4.4.3. Selectivity of OEs and (*E*)-Anethole for the Immature Stage

Thirty cards were used per treatment, each receiving approximately 125 *E. kuehniella* eggs containing the parasitoid in the egg–larva period (0–24 h of parasitism), and in the pre-pupa (72–96 h of parasitism) and pupa (168–192 h of parasitism) stages [84]. The cards with the host eggs were immersed in the treatment solutions, as mentioned in Section 4.4.1 for each EO for 5s. After evaporation of the solvent, each card was placed individually in glass tubes (8 cm high × 2.5 cm in diameter) and sealed with polyethylene film. A drop of pure honey was added to the wall of each tube to feed the emerged parasitoids.

The experiment was conducted in a completely randomized design with 30 biological replicates per treatment, with each replicate consisting of one card containing approximately 125 parasitized host eggs. The card was considered the experimental unit, whereas the individual eggs within each card were considered subsamples and were not treated as independent replicates. After parasitoid emergence, the numbers of parasitized eggs and emerged insects were recorded to calculate the emergence rate and the percentage reduction in the beneficial capacity of the parasitoid according to the IOBC methodology [42,43].

### 4.5. Analysis of the Data Obtained

The data on insect survival over time were analyzed using the nonparametric Kaplan–Meier estimator and subjected to the log-rank test using the survival package. Survival curves were compared using the pairwise multiple comparison test. The median lethal time (LT_50_) was estimated for each treatment (Section 2.2 and Section 2.3). To determine the lethal dose 50 (LD_50_), the data were subjected to Logit analysis using the drc package [92] (Ritz et al. 2015) (Section 2.3). These analyses were performed using the statistical program R^®^ version 4.0 [93].

Data analysis for the preparation of life tables was conducted using the TWOSEX-MSChart program version 2025.03.05 [94]. The means, variances, and standard errors of the demographic parameters were compared between treatments using the bootstrap method with 100,000 replicates [94]. The life table considers the means of the parameters of survival, life expectancy, and fertility up to age x and stage *j*. The differences between treatments were evaluated using the paired bootstrap test, with a significance level of 5%.

The experimental unit was defined as one individual female parasitoid in the adult assay and one card containing approximately 125 parasitized host eggs in the immature-stage assay. The individual eggs within each card were considered subsamples and were not treated as independent observations. Data on the number of parasitized eggs and emergence of *T. pretiosum* were submitted to Shapiro–Wilk and Bartlett tests to verify the assumptions of normality and homoscedasticity. As they did not assume normal distribution, they were adjusted to GLM (Generalized Linear Models) for the best distribution, Poisson or quasi-binomial, and the means were compared by contrast analysis. Data on the longevity of female *T. pretiosum* were submitted to Shapiro–Wilk and Bartlett tests to verify the assumptions of normality and homoscedasticity. As they did not assume normal distribution, they were analyzed using the nonparametric Kruskal–Wallis test (α = 0.05) from the Agricolae package, followed by pairwise comparisons of the rankings. Survival data over time were submitted to Weibull survival analysis using the Survival package. After selecting the most appropriate mathematical model through residual analysis, a contrast analysis was performed to verify the similarity between the treatments used to form congeneric groups. The percentage reduction (PR) in adult emergence was calculated using the formula: (1 − adult emergence in treatment/adult emergence in control) × 100. The classification of compounds was carried out in accordance with members of the International Organization for Biological Control (IOBC): class 1 = harmless (PR < 30%), class 2 = slightly harmful (30% ≤ PR < 80), class 3 = moderately harmful (80% ≤ PR ≤ 99), and class 4 = harmful (PR > 99%) (Section 2.6 and Section 2.7) [92,93].

## 5. Conclusions

The essential oils (EOs) of *F. vulgare* var. *dulce* and *S. aromaticum* were toxic to *S. frugiperda* (LD_50_ = 41 and 54.2 µg/caterpillar, respectively). In addition to causing acute mortality, both EOs induced sublethal effects by reducing larval development, fecundity, and key demographic parameters. (*E*)-anethole was identified as the major constituent underlying the insecticidal activity of *F. vulgare* var. *dulce* EO, whereas the isolated major constituents of *S. aromaticum* EO did not reproduce the toxicity of the complete EO, suggesting potential synergistic interactions among its constituents. Furthermore, *F. vulgare* var. *dulce* EO and (*E*)-anethole were selective toward *T. pretiosum* during the egg–larva period and pupal stage. Overall, these findings highlight the potential of these botanical products as sustainable alternatives for the integrated management of *S. frugiperda*. However, these conclusions are based on laboratory bioassays, and the reduced sample size obtained for reproductive parameters in the *F. vulgare* var. *dulce* treatment warrants cautious interpretation. Future research should prioritize semi-field and field trials to confirm the efficacy and selectivity of these EOs under practical application conditions, elucidate their biochemical mode of action in *S. frugiperda*, assess their impact on other natural enemies, and explore formulation strategies that improve the stability and field persistence of these botanical products.

## Figures and Tables

**Table 1:** Chemical composition of essential oils from *Foeniculum vulgare* var. *dulce* (Apiaceae) and *Syzygium aromaticum* (Myrtaceae).

Nº	Compound	RI^a^	RI^b^	Peak Area (%)^c^	
				*F. vulgare*	*S. aromaticum*
1	*α*-pinene	929	932	1.76	-
2	*β*-pinene	972	974	0.48	-
3	*β*-myrcene	986	988	0.55	-
4	*α*-phellandrene	1002	1002	1.94	-
5	*p*-cymene	1020	1020	1.46	-
6	limonene	1024	1024	4.51	-
7	eucalyptol (1,8-Cineole)	1026	1026	0.19	-
8	fenchone	1083	1083	4.31	-
9	NI	1194	-	0.84	-
10	*p*-anisaldehyde	1250	1247	0.61	-
11	(*E*)-anethole	1284	1282	83.35	-
12	eugenol	1357	1356	-	76.18
13	(*E*)-caryophyllene	1411	1417	-	19.9
14	NI	1419	-	-	0.2
15	aromandendrene	1440	1439	-	0.19
16	*α*-humulene	1444	1452	-	2.26
17	acetyl eugenol	1523	1521	-	1.02
Number of identified compounds				11	6
Total area (%)				100	99.75

RI^a^: retention indices calculated against n-alkanes series (C_8_-C_20_) on the column RTX-5MS by elution order; RI^b^: retention indices on an apolar column reported in the literature. Peak area (%)^c^: average of the relative percentage area of the chromatographic peaks. NI: not identified.

**Table 2:** Lethal dose 50 (LD_50_) and lethal dose 90 (LD_90_) of essential oils from *Syzygium aromaticum* (Myrtaceae) and *Foeniculum vulgare* var. *dulce* (Apiaceae), and of the active ingredient chlorpyrifos for *Spodoptera frugiperda* caterpillars.

Treatment	X^2^	df	LD_50_ (UL − LL) (μg/caterpillar)	LD_90_ (UL − LL) (μg/caterpillar)
*S. aromaticum*	462.7	498	54.2 (51.0–57.4)	92.6 (83.2–102.1)
*F. vulgare* var. *dulce*	452	498	41.0 (38.5–43.3)	70 (63.0–76.1)
Chlorpyrifos	452	498	0.03 (0.02–0.04)	0.3 (0.14–0.39)

Note: LD_50_ and LD_90_ were estimated using a two-parameter log-logistic model with lower and upper limits fixed at 0 and 1, respectively. CI = 95% confidence interval; χ^2^ = chi-square goodness-of-fit statistic; df = degrees of freedom. *N* = 500 larvae per treatment, corresponding to 100 larvae at each five tested concentrations; the untreated control was not included in the dose–response analysis.

**Table 3:** Mean (±SE) of the development time of the life stages and longevity of *Spodoptera frugiperda* exposed to essential oils from *Eugenia caryophyllus* (Myrtaceae) and *Foenicullum vulgare* var. *dulce* (Apiaceae), and chlorpyrifos, when 72-h-old caterpillars were treated.

**Development Time (Days)**	**Stages**	**Acetona**		***E. caryophyllus***		***F. vulgare* var. *dulce***		**Chlorpyrifos**	
		**N**	**Mean ± SE**	**N**	**Mean ± SE**	**N**	**Mean ± SE**	**N**	**Mean ± SE**
	Egg	100	2.00 ± 0.0 a	100	2.00 ± 0.00 a	100	2.00 ± 0.00 a	100	2.00 ± 0.00 a
	Cartepillar	95	14.37 ± 0.09 c	41	15.27 ± 0.28 b	24	17.00 ± 0.41 a	33	15.91 ± 0.48 b
	Pre-pupa	94	1.90 ± 0.06 a	40	1.82 ± 0.09 a	24	2.12 ± 0.16 a	33	1.76 ± 0.10 a
	Pupa	94	11.07 ± 0.12 a	36	11.25 ± 0.19 a	23	11.65 ± 0.31 a	31	10.97 ± 0.18 a
	Egg-pupa	94	38.25 ± 0.22 a	36	30.17 ± 0.27 c	23	32.48 ± 0.53 b	31	30.03 ± 0.30 c
Longevity (days)	Adult	94	9.18 ± 0.23 a	36	7.56 ± 0.43 b	23	7.65 ± 0.44 b	31	8.45 ± 0.33 a
Life cycle (days) *	Female	44	38.75 ± 0.33 a	13	38.15 ± 0.71 b	10	39.9 ± 0.41 a	15	38.73 ± 0.55 a
	Male	50	38.28 ± 0.30 b	23	37.48 ± 0.56 c	13	40.31 ± 0.54 a	16	38.25 ± 0.55 b
	Egg-adult	94	38.5 ± 0.22 b	36	37.72 ± 0.44 b	23	40.13 ± 0.35 a	31	38.48 ± 0.32 b

Means in the same line followed by different letters differ from each other (p < 0.05). Differences between treatments were obtained using the paired Bootstrap test with 100,000 replicates. N = number of specimens in each development phase. Average total life history for males and females in days, only for insects that became adults.

**Table 4:** Mean (±SE) reproductive parameters of adult *Spodoptera frugiperda* that developed from second-instar caterpillar exposed to the LD_50_ of plant essential oils and chlorpyrifos.

Parameter	Acetone		*Syzygium aromaticum*		*Foeniculum vulgare* Var. *dulce*		Chlorpyrifos	
	N	Mean ± SE	N	Mean ± SE	N	Mean ± SE	N	Mean ± SE
Total fertility rate	44	1252.7 ± 114.95 a	13	969.69 ± 191.94 b	10	473.7 ± 131.44 c	15	830.4 ± 161.58 b
Effective fertility (M/F) *	41	1344.37 ± 110.29 a	11	1146.0 ± 178.39 b	10	473.7 ± 131.44 c	13	958.15 ± 161.58 b
Egg-laying (days)	41	3.88 ± 0.25 a	11	3.37 ± 0.52 a	10	2.40 ± 0.48 b	13	3.31 ± 0.58 a
APOP (days)	41	3.32 ± 0.14 a	11	3.00± 0.40 a	10	3.30 ± 0.30 a	13	3.37 ± 0.39 a
TPOP (days)	41	31.88 ±0.20 b	11	32.27 ± 0.38 b	10	34.40 ± 0.95 a	13	32.54 ± 0.57 b
MDF (O/F)	-	2779	-	819	-	456	-	1005
MTF (O/F)	-	1557	-	1954	-	1479	-	2164

Maximum daily fertility (MDF), maximum total fertility (MTF), adult pre-oviposition period (APOP), and total pre-oviposition period (TPOP). * Total number of females that oviposited. Means in the same row followed by different letters are significantly different (*p* < 0.05). Differences between treatments were obtained using the paired Bootstrap test with 100,000 repetitions. (O/F) = eggs per female. N = number of specimens for each parameter evaluated.

**Table 5:** Mean (±SE) of demographic parameters of *Spodoptera frugiperda* obtained from second-instar caterpillars that were exposed to the LD_50_ of the treatments.

Demographic Parameter	Acetone	*Syzygium aromaticum*	*Foeniculum vulgare* Var. *dulce*	Chlorpyrifos
Intrinsic growth rate (r)	0.19 ± 0.01 a	0.14 ± 0.010 b	0.11 ± 0.010 c	0.14 ± 0.01 b
Finite rate of growth (λ)	1.20 ± 0.01 a	1.15 ± 0.01 b	1.11 ± 0.01 c	1.15 ± 0.01 b
Net Reproduction Rate (*R*0)	551.19 ± 70.82 a	126.06 ± 40.48 b	47.37 ± 18.93 b	124.56 ± 37.89 b
Average generation time (T)	33.857 ± 0.22 a	34.64 ± 0.42 a	36.19 ± 1.24 a	34.26 ± 0.49 a
Gross reproduction rate (*GRR*)	685.1 ± 93.64 a	445.31 ± 129.00 ab	324.66 ± 153.92 b	463.34 ± 133.67 ab

Means in the same row followed by different letters differ from each other (*p* < 0.05). Differences between treatments were obtained using the paired Bootstrap test with 100,000 repetitions. N = number of specimens at each developmental stage.

**Table 6:** Mean (±SE) longevity of adult females (days), number of parasitized eggs, emergence (%), emergence reduction (%), and IOBC toxicity classification of *Trichogramma pretiosum* females exposed to treated *Ephestia kuehniella* eggs.

Treatment	Female Longevity (Days)	Number of Parasitized Eggs	Emergence (%)	ER (%)	Class
Acetone	9.1 ± 1.6 a	18.2 ± 2.5 a	64.1 ± 8.5 a	-	
*F. vulgare* var. *dulce*	7.3 ± 3.8 ab	3.6 ± 1.0 c	12.1 ± 5.0 b	81.12	3
(*E*)-anethole	5.4 ± 4.2 b	6.0 ± 1.4 b	11.7 ± 5.0 b	81.7	3
*S. aromaticum*	-	0.5 ± 0.1 d	-	100	4
Chlorpyrifos	-	5.4 ± 0.5 b	-	100	4

Means followed by the same letter within a column are not significantly different (*p* > 0.05). Female longevity was analyzed using the Kruskal–Wallis test. Number of parasitized eggs was analyzed using a generalized linear model (GLM) with Poisson distribution. Emergence was analyzed using a GLM with quasibinomial distribution. Emergence reduction (ER) was calculated as (1 − emergence in treatment/emergence in control) × 100. Toxicity classes followed IOBC standards: Class 1, harmless (ER < 30%); Class 2, slightly harmful (30% ≤ ER < 80%); Class 3, moderately harmful (80% ≤ ER ≤ 99%), and Class 4, harmful (ER > 99%) [42,43].

**Table 7:** Mean (±SE) emergence (%), emergence reduction (%), and IOBC toxicity classification of *Trichogramma pretiosum* exposed during the egg–larva period, and prepupal and pupal stages to essential oils and their major compound.

Treatment	Egg–Larva			Prepupa			Pupa		
	Emergence (%)	ER (%)	Class	Emergence (%)	ER (%)	Class	Emergence (%)	ER (%)	Class
Acetone	67.3 ± 4.8 a	-	-	74.4 ± 6.5 a	-		78.2 ± 4.0 a	-	
*F. vulgare* var. *dulce*	50.1 ± 6.1 a	25.5	1	61.8 ± 6.2 a	16.9	1	51.2 ± 6.4 b	34.5	2
(*E*)-anethole	59.1 ± 5.7 a	12.2	1	60.2 ± 6.8 a	19.1	1	72.6 ± 5.4 a	7.2	1
*S. aromaticum*	9.8 ± 4.5 b	85.4	3	3.3 ± 1.3 b	95.6	3	3.1 ± 1.2 c	96.1	3
Chlorpyrifos	2.6 ± 1.9 b	96.1	3	3.4 ± 2.1 b	95.4	3	-	100	4

Means followed by the same letter within a column are not significantly different according to the F-test (GLM with quasibinomial distribution; *p* > 0.05). Emergence reduction (ER) = (1 − emergence in treatment/emergence in control) × 100. IOBC toxicity classes: Class 1, harmless; Class 2, slightly harmful; Class 3, moderately harmful, and Class 4, harmful [42,43].

## Data Availability

The original contributions presented in this study are included in the article. Further inquiries can be directed to the corresponding author.

## Abbreviations

The following abbreviations are used in this manuscript:

EOs	Essential oils
EO	Essential oil
GM	Genetically modified
IPM	Integrated pest management (IPM)
RI	Retention index
RT	Retention time
